# Artificial Intelligence-Enabled Exosomes in Precision Oncology: A Framework for Clinical Utility and Biomedical Applications

**DOI:** 10.3390/cimb48070704

**Published:** 2026-07-10

**Authors:** Prakash Gangadaran, Ramya Lakshmi Rajendran, Muthu Subash Kavitha, Byeong-Cheol Ahn

**Affiliations:** 1 Department of Nuclear Medicine, School of Medicine, Kyungpook National University, Daegu 41944, Republic of Korea; prakashg@knu.ac.kr (P.G.); ramyag@knu.ac.kr (R.L.R.); 2 Cardiovascular Research Institute, Kyungpook National University, Daegu 41944, Republic of Korea; 3 BK21 FOUR KNU Convergence Educational Program of Biomedical Sciences for Creative Future Talents, School of Medicine, Kyungpook National University, Daegu 41944, Republic of Korea; 4 School of Information and Data Sciences, Nagasaki University, Nagasaki 852-8521, Japan; kavitha@nagasaki-u.ac.jp; 5 Department of Nuclear Medicine, Kyungpook National University Hospital, Daegu 41944, Republic of Korea

**Keywords:** exosomes, artificial intelligence, precision oncology, biomarkers, liquid biopsy

## Abstract

Exosomes are 30–150 nm extracellular vesicles that convey molecular information reflecting the physiological and pathological states of their source cells. In precision oncology, they function as a non-invasive “liquid biopsy,” enabling real-time monitoring of tumor dynamics and metastasis. However, extreme biofluid heterogeneity poses significant challenges for their isolation and analysis using conventional statistical approaches. This review aims to examine how artificial intelligence (AI), specifically machine learning and deep learning, transforms complex exosomal “noise” into actionable clinical insights. AI enhances exosome isolation, enables disease-specific biomarker identification, and predicts therapeutic responses with high precision. Integrating multi-omics data and single-exosome analysis enables AI-driven models to facilitate early cancer detection and therapeutic resistance monitoring. Despite challenges related to standardization and data privacy, the convergence of AI and exosome biology is poised to transform reactive cancer treatments into a proactive, personalized medical ecosystem. This approach also provides a framework for managing other complex systemic diseases.

## 1. Introduction

Exosomes are 30–150 nm membrane-bound extracellular vesicles (EVs) now recognized as sophisticated biological messengers carrying a complex cargo of biomolecules, including proteins, nucleic acids, lipids, and metabolites [1,2,3]. More recently, they have emerged as promising nanoscale EVs that reflect the physiological and pathological states of their source cells. In oncology, they are increasingly used in liquid biopsy platforms to offer real-time insights into tumor biology and mechanisms of metastasis, among others [4,5]. Here, a ‘minimal-invasive or non-invasive liquid biopsy’ is formally defined as the isolation and molecular analysis of tumor-derived biomarkers such as circulating tumor cells, cell-free nucleic acids, or EVs directly from easily accessible biological fluids (e.g., blood, urine, or saliva) instead of solid tissue. Unlike traditional tissue biopsies, which are invasive, localized, and carry risks of clinical complications, exosomal liquid biopsies are minimally disruptive or completely non-invasive, allowing for repeatable, longitudinal monitoring of the entire systemic tumor landscape over time [6,7,8]. However, the release of billions of exosomes from diverse cellular sources presents significant analytical challenges that exceed the capabilities of conventional statistical approaches, thereby motivating the integration of artificial intelligence (AI) for their interpretation [9]. Machine learning (ML) approaches, particularly deep learning (DL) methods, offer the distinct advantage of identifying latent patterns in high-dimensional exosomal datasets that may remain undetectable without such approaches, thereby converting molecular noise into biologically meaningful signals [9].

Existing cancer diagnosis methodologies rely on predefined biomarkers, which are often insufficient to capture the complex and heterogeneous nature of cancer [4]. However, the AI-based analysis of exosomes enables simultaneous evaluation of thousands of parameters, identifying nonlinear relationships, and adapting the prediction models based on incoming data. This integrative interdisciplinary approach enables progress toward previously unattainable goals, serving as a proof-of-concept for early cancer detection in pre-symptomatic individuals, real-time monitoring of minimal disease, and prediction of therapeutic responses before treatment initiation, among others [9].

The clinical value of AI-based exosome analysis extends beyond oncology, encompassing a broad spectrum of conditions, including neurodegenerative diseases, cardiovascular conditions, autoimmune disorders, and infectious diseases [10,11,12,13]. While this review primarily focuses on precision oncology as the baseline paradigm for AI integration, these non-oncological applications are briefly highlighted to underscore how the computational framework developed for cancer can be adapted to other pathological states associated with altered exosome release. Tissue biopsies remain central to early cancer detection, while exosomal signatures represent highly heterogeneous but significant prognostic biomarkers whose analysis can be enhanced with AI-based strategies [12].

**Figure 1:**
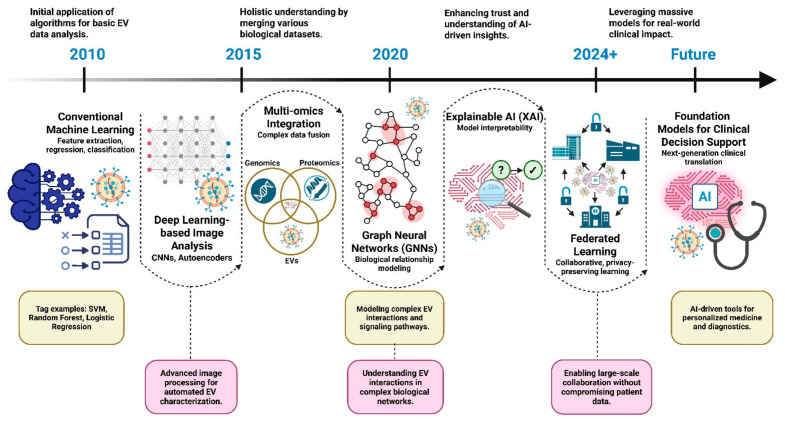
Historical roadmap of AI applications in extracellular vesicle (EV) research. The timeline illustrates the evolution of computational approaches from conventional machine learning (feature extraction, regression, and classification) to deep learning, multi-omics integration, graph neural networks (GNNs), explainable AI (XAI), federated learning, and future foundation models for clinical decision support, highlighting the progressive clinical translation of AI-enabled EV research. Created in BioRender. Gangadaran, P. (2026); https://BioRender.com/md2vyek (accessed on 30 June 2026).

This review aims to examine advanced ML and DL techniques for learning exosome signatures to better understand cancer pathology within the framework of precision oncology. By specifically focusing on these two computational subsets rather than broad AI concepts, we establish a precise analytical blueprint for interpreting exosomal multi-omics. By establishing precision oncology as our primary focal lens, we explore these advanced approaches to provide a foundational blueprint, while secondary consideration is given to broader medical applications to help establish a practical roadmap for translating this powerful technology into improved patient outcomes (Figure 1).

## 2. Biology and Characteristics of Exosomes

Exosomes originate from a tightly regulated intracellular pathway that differentiates them from other EVs. Their biogenesis begins with inward plasma membrane budding to form early endosomes, which subsequently mature into late endosomes or multivesicular bodies (MVBs) [4]. Intraluminal vesicles within MVBs eventually become exosomes following fusion with the plasma membrane [2,14]. This process is mediated via the endosomal sorting complex required for transport (ESCRT), comprising 20 different proteins grouped into four subcomplexes: ESCRT-0, -I, -II, and -III [1]. Recent studies suggest that exosome biogenesis can also occur via ESCRT-independent pathways, with tetraspanins (CD63, CD81, and CD9), ceramide lipids, and heat shock proteins playing key roles [3]. Exosome secretion and composition are dynamically regulated in response to different cellular stressors such as hypoxia, acidosis, and nutrient starvation, which are commonly observed in tumor microenvironments [4]. Cancer cells exhibit higher levels of exosome secretion compared to normal cells [5].

Exosomes function as molecular shuttles, selectively transporting various molecules according to their cellular origins. Proteomic analyses have identified over 4400 different proteins within exosomes. Exosomal proteins include membrane transport and fusion proteins such as Rabs, annexins, flotillin, tetraspanins, integrins, major histocompatibility complex components, and proteins involved in MVB biogenesis [1,3]. These proteins can provide insights into the cellular origins and functional roles of exosomes.

The nucleic acid content of exosomes is particularly valuable for developing diagnostic tools. Exosomes contain messenger RNA, microRNA (miRNA), long non-coding RNA, circular RNA, and DNA fragments, including mitochondrial and genomic DNA. Encapsulation within the lipid bilayer protects exosomal nucleic acids, providing stability and making exosomes ideal biomarker candidates [4,15,16]. The miRNA cargo of tumor-derived exosomes is highly valuable for distinguishing patients with malignancies from healthy individuals [17]. Furthermore, this encapsulated molecular cargo correlates closely with real-time disease progression and therapeutic response in patients undergoing active cancer treatment.

The lipid composition of exosomes is enriched in cholesterol, sphingomyelin, ceramide, and phosphatidylserine, which confer structural rigidity and enhance stability during circulation. These lipids also contribute to intercellular signaling and modulate the biological activities of exosomes [18,19].

Exosomes serve as key regulators of communication among tumor cells, stromal, immune, and endothelial cells [5]. Tumor-derived exosomes (TDEs) enhance angiogenesis through the transfer of pro-angiogenic signals such as VEGF, miR-9, and miR-210 to endothelial cells, thereby stimulating vascular network formation to support tumor growth [20,21,22].

TDEs modulate the tumor microenvironment in complex ways that suppress antitumor immunity and promote immune evasion through immunosuppressive mechanisms. TDEs transfer immunosuppressive signals—including PD-L1, TGF-β, and FasL—to tumor-infiltrating T cells, thereby inhibiting T-cell activation and inducing apoptosis in cytotoxic T cells [23]. TDEs also promote the proliferation and activation of myeloid-derived suppressor cells and regulatory T cells, thereby reinforcing an immunosuppressive tumor microenvironment that supports tumor progression [24].

A particularly notable feature of exosomes is their role in forming pre-metastatic niches at distant sites from the primary tumor. Hoshino et al. (2015) show that tumor-derived exosomes exhibit organ-specific biodistribution patterns depending on integrin expression profiles, thereby promoting pre-metastatic niche formation through recruiting bone marrow-derived cells and remodeling of the extracellular matrix at future metastatic sites [25].

## 3. Exosomes in Cancer Diagnosis and Prognosis

Tumor-derived exosomes, which are abundant in body fluids such as blood, urine, saliva, and cerebrospinal fluid, represent a promising approach for exploring the tumor microenvironment without the need for tissue biopsies. Protein-based exosomal markers such as glypican-1 (GPC1) show remarkable diagnostic performance for detecting early-stage pancreatic cancer, distinguishing affected patients from healthy individuals and benign pancreatic cohorts with an absolute sensitivity and specificity of 100% in initial validation sets (*p* < 0.0001) [26]. In lung cancer, exosomal miR-17-3p, miR-21, and miR-106a are present at significantly higher levels in patient sera than in healthy individuals, supporting early-stage disease detection in lung cancer detection, these serum exosomal miRNA signatures achieve a combined area under the receiver operating characteristic curve (AUC) ranging from 0.83 to 0.91, providing a statistically significant improvement over single-marker evaluations (*p* < 0.01) [27].

For instance, exosomal DNA harboring mutations such as KRAS and EGFR has been successfully detected in the plasma of patients with pancreatic cancer and with non-small-cell lung cancer (NSCLC), respectively, supporting the feasibility of non-invasive genetic profiling without the need for tumor biopsy [28,29]. Encapsulation within the exosomal lipid bilayer protects nucleic acids from degradation, thus increasing their stability compared to circulating cell-free DNA and RNA [16].

Exosomal biomarker signatures have been associated with cancer stage, metastatic potential, and therapeutic response. In breast cancer, high levels of exosome survivin are associated with advanced cancer stage and poorer clinical outcomes [30,31]. Similarly, in patients with melanoma, high levels of exosomal PD-L1 are associated with reduced responsiveness to anti-PD-1 immunotherapy [32].

Moreover, dynamic monitoring of exosomal biomarkers facilitates real-time evaluation of treatment efficacy and facilitates early detection of disease relapse. Patients with colorectal cancer who display decreased exosomal miR-21 biomarkers following surgery experience improved progression-free survival, whereas increased biomarkers are associated with disease relapses [33]. This dynamic monitoring of exosomal biomarkers highlights their advantages over conventional tissue-based biomarker analyses.

Exosome-based liquid biopsy technologies hold significant potential for minimally invasive cancer management. Unlike circulating tumor cells, which are rare, exosomes are highly abundant in the bloodstream of patients with cancer (10^9^–10^12^ particles/mL), making them a more reliable source for detection [34].

**Figure 2:**
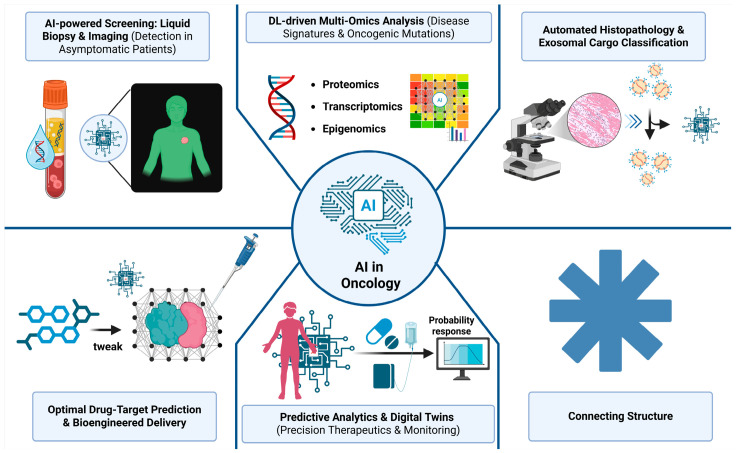
Multifaceted Applications of AI in Oncology. This diagram illustrates the diverse clinical and research areas where AI integration enhances cancer care. AI algorithms support tumor screening and early detection via identifying malignancies in individuals with no overt symptoms through liquid biopsy and imaging. In genomics and biomarker discovery, DL models analyze multi-omics datasets to reveal disease-specific signatures and oncogenic mutations. Microscope-based research employs AI for automated histopathology analysis and high-throughput characterization of exosomal cargo. Furthermore, AI accelerates drug design via predicting optimal drug–target interactions and bioengineered delivery modifications. These advances enable personalized medicines, where predictive analytics and digital twins guide therapeutic strategies and monitor real-time treatment responses. Abbreviations: AI, artificial intelligence; DL, deep learning. Created in BioRender. Gangadaran, P. (2026); https://BioRender.com/bhw08fm (accessed on 11 May 2026).

ExoSearch chip enables continuous-flow isolation and release of plasma exosomes from 10 μL to 10 mL samples from patients with ovarian cancer. Multiplexed detection of CA-125, EpCAM, and CD24 in ovarian cancer plasma showed excellent diagnostic performance (AUC = 1.0, *p* = 0.001) [35]. Despite these promising developments, the applicable scope of these platforms is strictly constrained by the limitations of their underlying isolation methods. For instance, while ultracentrifugation remains the historical benchmark for high-volume isolation, it suffers from low throughput, lipoprotein co-isolation, and structural damage to vesicles caused by high shear forces. Conversely, size-exclusion chromatography (SEC) preserves vesicle integrity and provides superior purity profiles, but heavily dilutes the sample, rendering rare-event biomarker identification difficult without secondary concentration. Microfluidic-based capture strategies offer exceptional sensitivity for low-volume clinical samples, yet they are limited by low processing throughput and a high susceptibility to channel clogging when handling viscous biofluids like whole plasma [36,37,38]. Current research efforts are focused on establishing standardized guidelines for exosome research through the International Society for Extracellular Vesicles (ISEV), thereby hastening the clinical translation of exosome-based diagnostics. Figure 2 and Table 1 provide an overview of the major AI applications in precision oncology, the key computational methodologies used in exosome research, and their current stages of clinical translation.

## 4. Artificial Intelligence in Cancer Research

Early detection and rapid cancer diagnosis remain major global challenges. Conventional cancer diagnosis techniques include clinical evaluation, imaging, and tissue biopsy. Presently, molecular diagnostics have been integrated alongside these techniques, enabling improved identification of genetic alterations associated with tumor initiation and progression. Major challenges associated with molecular diagnostic techniques include tissue heterogeneity and the evaluation and analysis of large-scale genomic datasets. These factors can delay data interpretation and, consequently, diagnosis. Integrating AI into these clinical workflows can effectively hasten the diagnostic process. AI approaches such as DL have been shown to improve accuracy in data analysis and interpretation, thereby supporting patient diagnosis and personalized treatment planning [39].

### 4.1. Fundamentals of Artificial Intelligence, Machine Learning, and Deep Learning

ML is a subset of AI that enables systems to identify patterns in data and make predictions without explicit programming. ML algorithms are generally classified into supervised, unsupervised, and reinforcement learning. DL is a subset of ML that employs multi-layered artificial neural networks to automatically extract hierarchical representations from raw data [40]. ML has become integral to modern technology, powering applications from web search algorithms and social media content filtering to personalized recommendations on e-commerce platforms [40]. DL is a subset of ML that employs multi-layered artificial neural networks to automatically extract hierarchical representations from raw data. Key DL architectures include convolutional neural networks (CNNs), recurrent neural networks, and transformers, which have enabled major advances in computer vision, natural language processing, and autonomous systems [41,42].

### 4.2. Artificial Intelligence Applications in Oncology

AI is transforming oncology through multifaceted applications in cancer detection, genomic analysis, treatment optimization, drug discovery, and prognostic prediction. DL models have demonstrated radiologist-level accuracy in interpreting mammograms and computed tomography (CT) scans for lung cancer screening, while also enabling automated histopathological analysis and CT scans for lung cancer screening [43,44]. AI-driven genomic approaches facilitate precision oncology via identifying oncogenic mutations and predicting therapeutic responses [45,46]. AI-powered radiomics enhances treatment planning for radiotherapy optimization [47,48] and surgical guidance. In drug development, AI accelerates discovery and repurposing through advanced predictive modeling [49,50]. Prognostic applications utilize AI to analyze longitudinal data for survival prediction and recurrence monitoring [9,51], collectively advancing personalized cancer care [52].

## 5. Integrating Artificial Intelligence with Exosome Research

AI is advancing exosome research via enhancing exosome isolation, characterization, biomarker discovery, and therapeutic applications. AI-driven algorithms optimize exosome isolation techniques via analyzing complex datasets from ultracentrifugation, microfluidics, and SEC, enhancing yield and purity [53]. ML models, such as support vector machines (SVMs) and deep neural networks, enable high-throughput characterization of exosomal cargo, including proteins, miRNAs, and lipids, which enables disease-specific biomarker identification. AI-driven predictive analytics integrate multi-omics datasets to reveal exosome-mediated signaling pathways and their contributions to cancer progression and immune modulation. Additionally, AI accelerates the development of exosome-based drug delivery systems via predicting optimal surface modifications and targeting efficiency [11,54,55]. Collectively, these advancements enhance exosome research, offering novel insights into their diagnostic and therapeutic potential.

The selection of specific AI architecture is not a one-size-fits-all decision; rather, it must be carefully aligned with the quantitative and qualitative constraints of the exosomal input data. While CNNs excel at parsing structural, spatial, and morphological information derived from nano-optical microfluidic devices, they are structurally ill-suited for processing tabular multi-omic expression counts. For instance, handling high-dimensional, low-sample-size arrays generated by SEC requires simpler, regularized architectures like SVMs or random forests to effectively suppress overfitting. Conversely, capturing nonlinear biological signaling dynamics necessitates the network-mapping capabilities of GNNs, though this comes at the cost of significant computational overhead and a reliance on extensively annotated prior literature graphs.

### 5.1. Artificial Intelligence for Exosome Biomarker Discovery

AI is transforming exosome biomarker discovery via leveraging advanced ML and DL algorithms to analyze complex exosomal cargos such as proteins, miRNAs, lipids, and nucleic acids with unprecedented precision and efficiency. AI-driven approaches enable disease-specific biomarker identification via integrating multi-omics data from exosome-derived components, enabling the detection of subtle patterns indicative of pathological states such as cancer, neurodegenerative disorders, and cardiovascular diseases [11,12,13,56]. For instance, SVMs and random forest classifiers have been used to distinguish exosomal miRNA profiles associated with breast cancer and glioblastoma, offering non-invasive diagnostic tools with high sensitivity and specificity [32]. Furthermore, AI enhances the predictive power of exosome-based liquid biopsies via correlating exosomal biomarkers with clinical outcomes, thereby enhancing prognostic accuracy and informing therapeutic decision-making [57]. Another study that used seven supervised ML platforms included 334 patients with epithelial ovarian cancer (EOC) and 101 patients with benign ovarian tumors in the study. ML models utilizing routine pretreatment blood parameters and age demonstrated superior performance compared to conventional statistical methods in predicting EOC and its associated clinical characteristics. Ensemble algorithms, particularly Random Forest, achieved excellent diagnostic accuracy, with an AUC of 0.968 and 92.4% accuracy in distinguishing EOC from benign ovarian tumors [58]. DL models, such as CNNs, have been applied to analyze high-throughput proteomic and lipidomic datasets, revealing novel exosomal signatures associated with disease progression and treatment response [59]. Additionally, AI accelerates the validation of potential biomarkers through automated feature selection and dimensionality reduction techniques, streamlining the transition from experimental discovery to clinical application. These AI-driven advancements not only enhance the understanding of exosome-mediated intercellular communication but also enable the development of minimally invasive, exosome-based diagnostic platforms, ultimately advancing precision medicine.

### 5.2. Artificial Intelligence-Based Classification and Cancer Detection

AI is transforming cancer detection and classification through advanced ML and DL algorithms to analyze complex data from radiological imaging, histopathology, and multi-omics platforms with high accuracy and efficiency. CNNs demonstrate radiologist-level performance in interpreting mammograms for breast cancer detection [43] and low-dose CT scans for lung cancer screening [44], reducing diagnostic errors and enabling early detection. In histopathology, AI automates whole-slide image analysis, accurately classifying tumor subtypes, grading malignancies, and identifying metastatic regions, thereby enhancing diagnostic precision and reducing interobserver variability [60]. AI-powered radiomics platforms extract quantitative features from medical imaging data to classify tumor phenotypes and predict treatment responses [47]. Additionally, AI integrates genomic, transcriptomic, and proteomic data to classify cancer types, identify driver mutations, and predict therapeutic targets, advancing precision oncology [46]. For example, AI models have been used to classify skin cancer at dermatologist-level accuracy using dermoscopic images [61]. These AI-driven innovations enhance diagnostic accuracy, streamline clinical workflows, reduce healthcare costs, and improve patient outcomes, establishing AI as a cornerstone of modern cancer care.

### 5.3. Artificial Intelligence for Predicting Therapeutic Response

AI is revolutionizing therapeutic response prediction in oncology by leveraging DL algorithms to analyze complex multimodal data, enabling personalized treatment strategies and improving patient outcomes. AI models integrate genomic profiles, radiomic features, clinical parameters, and digital pathology data to predict responses to chemotherapy, immunotherapy, targeted therapy, and radiation with high accuracy [62]. For instance, DL-based radiomics models predict neoadjuvant chemotherapy response in patients with breast cancer by analyzing dynamic contrast-enhanced MRI features [63], while ML algorithms utilizing whole-slide histopathology images accurately forecast immunotherapy outcomes in patients with advanced melanoma [64]. AI-powered platforms also analyze circulating tumor DNA and exosomal biomarkers to monitor real-time treatment response and detect emerging resistance mechanisms. Furthermore, GNNs have emerged as powerful tools for decoding the spatial organization of the tumor microenvironment beyond conventional cell density and proximity analyses. In NSCLC, GNN-based modeling of multiplex immunofluorescence data accurately predicted patient survival and revealed that context-dependent interactions among CD8^+^ T cells, PD-L1^+^ immune cells, and FOXP3^+^ regulatory T cells are major determinants of clinical outcome. These findings highlight the potential of spatial AI to identify clinically relevant biomarkers for precision oncology [65]. These AI-driven approaches, particularly DL-based computer vision, have revolutionized medical imaging by enabling accurate disease detection, diagnosis, and clinical decision-making. These technologies are increasingly integrated into clinical workflows, improving diagnostic precision and healthcare delivery [52].

## 6. Emerging Technologies and Multi-Omics Integration

The translation of exosome research from bench to bedside depends on the ability to process high-dimensional liquid biopsy data. Beyond simple quantification, the synergy between AI and multi-omics transforms biological “noise” into actionable clinical intelligence, redefining precision oncology [66]. Exosome-based diagnostics face significant challenges due to substantial biological and technical “exosomal noise,” which arises from background EVs, isolation-related artifacts, and high-dimensional multi-omics data [67,68]. We believe that AI-driven computational approaches may address these limitations by deconvolving complex datasets, reducing noise, and extracting clinically relevant exosomal signatures to enable accurate disease detection and prognosis.

**Figure 3:**
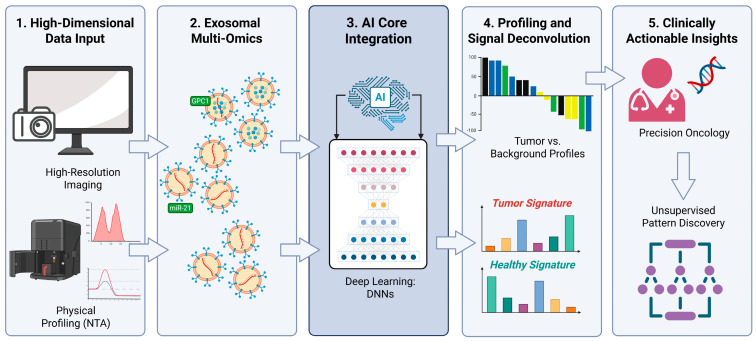
AI-Driven Analysis of Exosomal Multi-Omics. This schematic illustrates a computational pipeline used to transform high-dimensional biological data into clinically actionable insights. The process begins with high-dimensional data input, where high-resolution imaging and physical profiling, such as NTA, characterize exosome morphology and size distribution. Exosomes act as multi-omics carriers, transporting proteins (e.g., GPC1) and nucleic acids, including miRNA (e.g., miR-21), that reflect the state of their source cells. The AI core integrates these data using DL architectures, like deep neural networks. Through advanced profiling and deconvolution, the system identifies distinct exosome signatures and separates tumor-derived signals from healthy background vesicles, ultimately enabling unsupervised pattern discovery for precision oncology. **Abbreviations**: NTA, nanoparticle tracking analysis; GPC1, glypican-1; miRNA, microRNA; miR-21, microRNA-21; AI, artificial intelligence. Created in BioRender. Gangadaran, P. (2026); https://BioRender.com/8nkhlbv (accessed on 11 May 2026).

The primary challenge is the heterogeneity of biofluids; tumor-derived exosomes are often limited due to abundant healthy EVs. AI provides a computational framework to “deconvolve” these mixtures, identifying subtle multi-marker signatures missed by traditional statistical methods. Integrating genomic, proteomic, and lipidomic data enables AI-driven models to shift oncology from diagnosis to prediction, enabling real-time monitoring of tumor evolution and early detection of therapeutic resistance before it appears on standard imaging [69] (Figure 3).

Table 2 outlines the applications, technical constraints, and major limitations of key AI and analytical methodologies used in exosome research.

### 6.1. Artificial Intelligence-Driven Exosome Profiling

The primary challenge in exosome research remains the extreme heterogeneity of biofluids; a single milliliter of blood contains billions of EVs, only a small fraction of which are tumor-derived. To address this, AI-driven profiling utilizes DL architectures, such as CNNs and autoencoders, to identify the “molecular fingerprint” of malignant exosomes amid abundant healthy vesicles. Compared with traditional manual gating in flow cytometry, AI enables automated feature extraction by simultaneously analyzing hundreds of surface markers and morphological features, including size, density, and cargo to accurately classify exosome subpopulations [54,70]. Furthermore, ML models such as random forests and SVMs facilitate signature discovery by reducing thousands of variables to minimal biomarker panels. Selecting the most predictive miRNAs or proteins enables these tools to develop highly specific, cost-effective diagnostic assays suitable for clinical implementation [71].

### 6.2. Integration with Genomics, Proteomics, and Metabolomics

Exosomes act as “multi-omic carriers,” encapsulating DNA, RNA, proteins, and lipids that reflect the real-time physiological state of their cell of origin. In this context, AI integrates particularly ML and DL are transforming precision oncology by integrating multi-omics datasets into clinically actionable insights for improved cancer diagnosis, prognosis, and therapeutic decision-making. Advanced AI frameworks enable the analysis of complex biological data, accelerating biomarker discovery and supporting personalized cancer management [72].

### 6.3. Single-Exosome Analysis and Advanced Computational Tools

The future of precision oncology lies in the granular detail of single-exosome analysis (SEA), a paradigm shift influenced by the limitations of bulk analysis, which often masks rare, aggressive, drug-resistant exosomes that drive systemic metastasis. To achieve this resolution, computational nanoscopy uses advanced imaging tools, such as AI-enhanced nanoparticle tracking analysis, to determine the refractive index and biochemical composition of individual vesicles in real time. In clinical settings where single-exosome isolation remains challenging, digital deconvolution utilizes AI-powered algorithms to “mathematically untangle” bulk exosomal data. This approach allows researchers to estimate proportions of different cell-of-origin types, distinguishing exosomes derived from primary lung tumors from those originating from metastatic sites such as bone lesions [73]. Furthermore, integrating predictive analytics shifts the clinical focus from static diagnosis to dynamic treatment monitoring. Analyzing longitudinal changes in exosomal cargo during chemotherapy enables AI models to act as a sensitive “early warning system,” detecting molecular drug resistance often weeks before structural changes appear on traditional CT scans [70].

### 6.4. Cross-Methodological Comparison of Isolation and Computational Workflows

To provide a practical roadmap for clinical translation, it is necessary to contrast how typical physical isolation methods interface with downstream AI architectures. The choice of isolation methodology dictates the structural integrity, yield, and purity of the exosomal cargo [74,75], which directly establishes the type of high-dimensional data input available for ML or DL models.

## 7. Clinical Translation and Challenges

Despite the robust analytical capabilities of AI in exosome research, several systemic barriers must be addressed before these technologies can transition from specialized laboratories to routine clinical practice. The current “translational gap” arises from limited infrastructure to support high-throughput, standardized, and ethically sound processing of biological big data. For AI-driven exosomal diagnostics to succeed, the focus must shift from discovery-based research to implementation science, ensuring these models are accurate in controlled settings and reliable and reproducible across diverse, real-world patient populations.

### 7.1. Standardization of Exosome Isolation and Analysis

The lack of a gold standard for exosome isolation remains a major barrier to clinical reproducibility. Techniques such as ultracentrifugation, SEC, and microfluidic capture produce EVs with varying concentrations and purities [76], introducing technical variability that can lead to “garbage in, garbage out” outcomes in AI models. Clinical translation requires rigid adherence to standardized protocols (e.g., MISEV guidelines of ISEV) to eliminate preanalytical variability. Crucially, actionable translation demands locked preanalytical standard operating procedures (SOPs). This includes establishing strict time-to-centrifugation thresholds (<2 h post-phlebotomy), mandatory use of specific anti-coagulants (K2-EDTA preferred over heparin due to polymer chain reaction interference), and verified exclusion of large-vesicle contamination via computerized physical profiling [67,77]. AI algorithms must be deployed as gatekeepers to evaluate sample quality, automatically flagging and rejecting multi-omics inputs where the background nanoparticle-to-protein ratio falls outside preset analytical baseline ranges [78,79,80].

### 7.2. Data Quality and Model Validation

For an AI model to be clinically actionable, it must demonstrate robust generalizability beyond current models, often trained on small, single-center cohorts. Such narrow datasets increase the risk of overfitting, in which an algorithm achieves near-perfect accuracy on its training data but fails to generalize to the biological variability of real-world, diverse patient populations [81]. To mitigate this, clinical transition requires a tiered validation framework, and Explainable AI (XAI) frameworks must provide actionable outputs. Rather than a solitary probability score, XAI tools must generate feature-attribution maps (such as SHAP or LIME values) that explicitly link algorithmic calls back to individual, verifiable biological components such as specific surface proteins (e.g., GPC1) or miRNA targets (e.g., miR-21) [82]. This creates an explicit biological audit trail that clinicians can cross-reference with standard oncology benchmarks.

### 7.3. Ethical, Regulatory, and Data Privacy Considerations

Integrating AI with exosome-based multi-omics introduces significant ethical and legal complexities beyond traditional diagnostics. Given that exosomes carry diverse genetic material, they constitute highly sensitive patient data that could be used to re-identify individuals or predict secondary health risks, such as hereditary conditions or disease predispositions outside the scope of a primary cancer diagnosis. To address data privacy concerns, the field is shifting toward decentralized approaches such as Federated Learning. This approach allows AI models to be trained across multiple global institutions using local datasets without sharing raw patient information, thereby preserving confidentiality while leveraging large-scale data diversity [83]. Simultaneously, evolving regulatory pathways remain a critical bottleneck as major bodies such as the U.S. Food and Drug Administration (FDA) and European Medicines Agency (EMA) refine frameworks for Software as a Medical Device (SaMD). To secure regulatory approval, developers must establish robust evidentiary standards. This includes demonstrating clinical validity via rigorous bias assessment—such as performing stratified sub-analyses to confirm that diagnostic sensitivity and specificity remain equitable across ethnic, age, and gender sub-cohorts. Prospective clinical evaluation must be executed, moving beyond retrospective biobank analysis to demonstrate that the AI-exosome platform provides a statistically significant improvement in real-time patient management or clinical endpoints over existing standard-of-care diagnostics [71].

### 7.4. Mapping the Translational Spectrum: From Bench to Bedside

To accurately assess the horizon of AI-enabled exosome applications, it is critical to separate academic milestones from clinical readiness. The field can be mapped across five distinct operational phases:

Experimental & Discovery Phase: Single-exosome analysis (SEA) and DL-based sorting of sub-population cargo (e.g., tracing tissues of origin) remain firmly experimental. These platforms require specialized nanoscopy and specialized computational power not found in hospital laboratories. Preclinical Engineering: AI-guided surface modification for smart exosomal drug delivery (e.g., BBB-crossing or CRISPR cargo loading) is strictly preclinical. These models operate in simulated environments or animal models and face unresolved scaling, manufacturing, and safety checkpoints. Analytical Validation: Microfluidic chip platforms (such as ExoChip or ExoScreen) have achieved notable analytical metrics, demonstrating up to 90–97% sensitivity in specific cohorts. However, these are largely restricted to small, retrospective, single-center cohorts that are highly susceptible to overfitting. Clinical Validation: Multi-omic signatures analyzed by SVMs or Random Forests for common malignancies (e.g., lung or breast cancer tracking) are moving into early-stage clinical validation. They are proving useful as exploratory endpoints in longitudinal studies but do not yet replace tissue biopsies as primary diagnostic tools. Regulatory Approval: Currently, no fully AI-integrated exosomal liquid biopsy platform holds routine, wide-scale clearance from major international regulatory bodies (such as the FDA or EMA) for standalone primary diagnostic utility. Evolving software frameworks (like Software as a Medical Device—SaMD) remain an active hurdle.

## 8. Future Perspectives

Exosome research in precision oncology is shifting from passive observation to active intervention, driven by advances in AI and nanotechnology. Over the next decade, three transformative, autonomous therapeutics, digital twin integration, and multi-modal foundation models are expected to reshape the clinical landscape.

### 8.1. From Diagnostics to Artificial Intelligence-Guided “Smart” Therapeutics

While exosomes have primarily been viewed as diagnostic biomarkers, their future lies in their role as bioengineered delivery vehicles. AI is currently being used to optimize the loading of these vesicles with CRISPR-Cas9 components, RNA interference molecules, or chemotherapeutic agents. Future AI models will likely function as molecular architects, predicting precise surface modifications that enable engineered exosomes to bypass the blood–brain barrier or selectively target hypoxic tumor niches with surgical precision. This shift will move oncology away from systemic toxicity toward a “find-and-fix” paradigm, in which the same vesicle used for diagnosis also delivers the cure [66,69].

### 8.2. Rise of the “Exosome Digital Twin”

The most ambitious clinical frontier lies in the development of oncology “digital twins.” These platforms function as dynamic virtual replicas updated with longitudinal multi-omics data reflecting a patient’s unique tumor biology that evolves in real time.

Continuously integrating longitudinal exosomal data (genomic, proteomic, and metabolic changes) enables these AI-driven simulations to predict how cancer in a specific patient will respond to treatment “what-if” scenarios. Rather than relying on trial and error, clinicians will be able to test immunotherapies or targeted drugs on the digital twin, identifying the optimal therapeutic window and dosing schedule before the patient is administered any medication [71,84].

### 8.3. Multi-Modal Foundation Models and Global Equity

The next generation of AI in oncology will likely be driven by Foundation Models—large-scale architectures that process multimodal data, including exosomal profiles, radiology images, and pathology slides, simultaneously. These models will move the field beyond niche discoveries toward a unified understanding of cancer biology. Furthermore, as microfluidic lab-on-a-chip technologies become more affordable, AI-driven exosome analysis could democratize precision medicine. Providing high diagnostic accuracy through non-invasive methods may enable these tools to bring world-class oncology insights to resource-limited settings, narrowing the global gap in cancer survival rates [85,86].

## 9. Conclusions

Integrating AI into exosome research represents a watershed moment in precision oncology. Once dismissed as cellular waste, exosomes are now recognized as high-dimensional data carriers that capture the real-time evolution of the tumor microenvironment. As this review shows, the complexity of exosomal multi-omics spanning genomics, proteomics, and lipidomics, requires the computational rigor of AI to move beyond simple biomarker discovery toward a “find-and-understand” diagnostic paradigm. Leveraging DL for automated feature extraction and SEA enables us to begin to unravel liquid biopsy heterogeneity, identifying rare-event vesicles that drive metastasis and therapeutic resistance before clinical manifestation.

However, the path from bench to bedside remains constrained by the dual challenges of standardization and interpretability. The success of AI-driven exosome diagnostics depends on algorithmic accuracy as well as widespread adoption of rigorous isolation protocols and the development of XAI frameworks that provide clinicians with transparent, biologically grounded insights. Furthermore, as we navigate the ethical and regulatory complexities of genetic data privacy, the shift to decentralized models such as federated learning will be essential to ensure these innovations are secure and equitable.

Looking forward, the convergence of AI and exosome biology promises to shift oncology from reactive, “one-size-fits-all” treatments to a proactive, predictive, and personalized approach. Through patient-specific digital twins or AI-optimized exosomal delivery vehicles, the synergy of these fields is poised to redefine the boundaries of cancer care. Ultimately, the transition of exosomes from biological curiosities to clinical cornerstones remains an active multi-year endeavor. It depends not only on our ability to transform high-dimensional biological noise into actionable intelligence, but also on executing the prospective multi-center trials and rigorous analytical standardization required to move these platforms out of the discovery phase into routine clinical practice.

## Figures and Tables

**Table 1:** AI in Exosome Research: Applications and Technologies.

Focus Area	Description	Key AI/Computational Tools	Clinical Value & Translational Status
Biomarker Discovery	Identifying disease-specific exosomal proteins, miRNAs, and lipids	SVMs and random forests	Discovery stage: High-sensitivity multi-marker profiling; clinical validation still needed.
Cancer Classification	Tumor subtype classification and grading of malignancies	CNNs and deep neural networks	Early clinical stage: Improves diagnostic accuracy and consistency.
Therapeutic Prediction	Forecasting patient response to chemotherapy, immunotherapy, and radiation therapy	GNNs and DL-based radiomics	Proof-of-concept stage: Supports treatment stratification; requires prospective validation.
Exosome Profiling	Single–vesicle analysis to identify rare aggressive or drug-resistant exosomal subpopulations	Autoencoders, digital deconvolution algorithms	Preclinical stage: Enables early detection of molecular heterogeneity.
Drug Delivery	Engineering bioengineered vesicles for targeted therapy	AI-driven predictive modeling for surface modification	Preclinical stage: Enhances targeting efficiency; translation challenges remain.

Abbreviations: SVMs, support vector machines; CNN, convolutional neural networks; GNN, graph neural networks; DL, deep learning; AI, artificial intelligence.

**Table 2:** Scope, Application Boundaries, and Limitations of Core Methodologies.

Methodology/AI Tool	Main Applications	Technical Constraints	Key Limitations
CNNs	Image feature extraction; exosome morphology classification	Requires large, annotated image datasets; sensitive to imaging variations	Poor interpretability (“black box”); limited cross-platform robustness
GNNs	Multi-omics integration; tumor–immune network modeling	Relies on existing databases (KEGG, Reactome); high computational cost	Cannot easily handle novel molecules; over-smoothing in deep models
Autoencoders & Dimensionality Reduction	Pattern discovery; noise reduction; signal deconvolution	Requires large datasets	May lose rare but biologically important features
Single-Exosome Analysis (SEA)	Identification of rare and drug-resistant exosome subpopulations	Requires specialized instruments; low throughput	Sensitive to technical noise, sample loss, and contamination

Abbreviations: CNNs, convolutional neural networks; GNNs, graph neural networks; SEA, single-exosome analysis; KEGG, Kyoto Encyclopedia of Genes and Genomes.

## Data Availability

No new data were created or analyzed in this study.

## References

[1] Colombo M., Raposo G., Théry C. (2014). Biogenesis, Secretion, and Intercellular Interactions of Exosomes and Other Extracellular Vesicles. Annu. Rev. Cell Dev. Biol..

[2] Théry C., Witwer K.W., Aikawa E., Alcaraz M.J., Anderson J.D., Andriantsitohaina R., Antoniou A., Arab T., Archer F., Atkin-Smith G.K. (2018). Minimal Information for Studies of Extracellular Vesicles 2018 (MISEV2018): A Position Statement of the International Society for Extracellular Vesicles and Update of the MISEV2014 Guidelines. J. Extracell. Vesicles.

[3] Stuffers S., Sem Wegner C., Stenmark H., Brech A. (2009). Multivesicular Endosome Biogenesis in the Absence of ESCRTs. Traffic.

[4] Kalluri R., LeBleu V.S. (2020). The Biology, Function, and Biomedical Applications of Exosomes. Science.

[5] Thakur B.K., Zhang H., Becker A., Matei I., Huang Y., Costa-Silva B., Zheng Y., Hoshino A., Brazier H., Xiang J. (2014). Double-Stranded DNA in Exosomes: A Novel Biomarker in Cancer Detection. Cell Res..

[6] Ma K., Sun X., Liang C., Yi M., Shen L., Zhang J., Wang Z. (2026). Biosensors Based on Liquid Biopsy for Clinical Cancer Diagnosis. Biosens. Bioelectron..

[7] Ma L., Guo H., Zhao Y., Liu Z., Wang C., Bu J., Sun T., Wei J. (2024). Liquid Biopsy in Cancer: Current Status, Challenges and Future Prospects. Signal Transduct. Target. Ther..

[8] Yu W., Hurley J., Roberts D., Chakrabortty S.K., Enderle D., Noerholm M., Breakefield X.O., Skog J.K. (2021). Exosome-Based Liquid Biopsies in Cancer: Opportunities and Challenges. Ann. Oncol..

[9] Esteva A., Robicquet A., Ramsundar B., Kuleshov V., DePristo M., Chou K., Cui C., Corrado G., Thrun S., Dean J. (2019). A Guide to Deep Learning in Healthcare. Nat. Med..

[10] Mori M., Yoshinaga S., Moriyama T., Maekawa T. (2026). Infectious Disease Diagnosis by Artificial Intelligence (AI): Differences in Patient Backgrounds and Symptoms between Antigen Test Positives and Novel AI-Powered Pharyngeal Endoscopy Test Positives. PLoS Digit. Health.

[11] Dinc R., Ardic N. (2026). Artificial Intelligence for Exosomal Biomarker Discovery for Cardiovascular Diseases: Multi-Omics Integration, Reproducibility, and Translational Prospects. Cells.

[12] Picchio V., Pontecorvi V., Dhori X., Bordin A., Floris E., Cozzolino C., Frati G., Pagano F., Chimenti I., De Falco E. (2025). The Emerging Role of Artificial Intelligence Applied to Exosome Analysis: From Cancer Biology to Other Biomedical Fields. Life Sci..

[13] Zhu Q., Wu S., Huang P., Sun Q., Liu Z., Zhu X., Lee L.P., Liu F. (2026). AI-Driven Biomarker Learning for the Early Diagnosis of Neurodegenerative Diseases: ABLEDx. J. Nanobiotechnol..

[14] Rajendran R.L., Onkar A., Batabyal R., Jha S.K., Mahajan A.A., Koley M., Banerjee S., P A., Saha M., Ghosh S. (2026). Stem Cell-Derived Extracellular Vesicles and Artificial Nanovesicles: A Translational Framework for Cell-Free Wound Repair. npj Regen. Med..

[15] Skotland T., Sandvig K., Llorente A. (2017). Lipids in Exosomes: Current Knowledge and the Way Forward. Prog. Lipid Res..

[16] Record M., Carayon K., Poirot M., Silvente-Poirot S. (2014). Exosomes as New Vesicular Lipid Transporters Involved in Cell–Cell Communication and Various Pathophysiologies. Biochim. Biophys. Acta BBA-Mol. Cell Biol. Lipids.

[17] Padda J., Khalid K., Khedr A., Patel V., Al-Ewaidat O.A., Tasnim F., Padda S., Cooper A.C., Jean-Charles G. (2021). Exosome-Derived microRNA: Efficacy in Cancer. Cureus.

[18] Boilard E. (2018). Thematic Review Series: Exosomes and Microvesicles: Lipids as Key Components of Their Biogenesis and Functions Extracellular Vesicles and Their Content in Bioactive Lipid Mediators: More than a Sack of microRNA. J. Lipid Res..

[19] Ghadami S., Dellinger K. (2023). The Lipid Composition of Extracellular Vesicles: Applications in Diagnostics and Therapeutic Delivery. Front. Mol. Biosci..

[20] Zhuang G., Wu X., Jiang Z., Kasman I., Yao J., Guan Y., Oeh J., Modrusan Z., Bais C., Sampath D. (2012). Tumour-Secreted miR-9 Promotes Endothelial Cell Migration and Angiogenesis by Activating the JAK-STAT Pathway. EMBO J..

[21] Song W., Yan D., Wei T., Liu Q., Zhou X., Liu J. (2018). Tumor-Derived Extracellular Vesicles in Angiogenesis. Biomed. Pharmacother..

[22] Chen S., Sun J., Zhou H., Lei H., Zang D., Chen J. (2024). New Roles of Tumor-Derived Exosomes in Tumor Microenvironment. Chin. J. Cancer Res..

[23] Tang Q., Yang S., He G., Zheng H., Zhang S., Liu J., Wei S., Fan Q., Peng X., Li X. (2022). Tumor-Derived Exosomes in the Cancer Immune Microenvironment and Cancer Immunotherapy. Cancer Lett..

[24] Zhang W., Jin C., Liu S., Wan X., Li Y., Liu J., Duan Z., Ma J., Gao Y. (2026). Myeloid-Derived Suppressor Cells and Regulatory T Cells in Colorectal Cancer: A Synergistic Immunosuppressive Axis and Emerging Therapeutic Opportunities. Front. Immunol..

[25] Hoshino A., Costa-Silva B., Shen T.-L., Rodrigues G., Hashimoto A., Tesic Mark M., Molina H., Kohsaka S., Di Giannatale A., Ceder S. (2015). Tumour Exosome Integrins Determine Organotropic Metastasis. Nature.

[26] Melo S.A., Luecke L.B., Kahlert C., Fernandez A.F., Gammon S.T., Kaye J., LeBleu V.S., Mittendorf E.A., Weitz J., Rahbari N. (2015). Glypican-1 Identifies Cancer Exosomes and Detects Early Pancreatic Cancer. Nature.

[27] Rabinowits G., Gerçel-Taylor C., Day J.M., Taylor D.D., Kloecker G.H. (2009). Exosomal microRNA: A Diagnostic Marker for Lung Cancer. Clin. Lung Cancer.

[28] Castellanos-Rizaldos E., Zhang X., Tadigotla V.R., Grimm D.G., Karlovich C., Raez L.E., Skog J.K. (2019). Exosome-Based Detection of Activating and Resistance EGFR Mutations from Plasma of Non-Small Cell Lung Cancer Patients. Oncotarget.

[29] Nouri M., Sharif S., Soltani E., Mozaffari-Jovin S., Abbaszadegan M.R. (2025). Performance of KRAS Mutation Detection in Plasma Exosomes from Patients with Early-Stage Colorectal Cancer. J. Transl. Med..

[30] Khan S., Jutzy J.M.S., Aspe J.R., McGregor D.W., Neidigh J.W., Wall N.R. (2011). Survivin Is Released from Cancer Cells via Exosomes. Apoptosis.

[31] Halvaei S., Daryani S., Eslami-S Z., Samadi T., Jafarbeik-Iravani N., Bakhshayesh T.O., Majidzadeh-A K., Esmaeili R. (2018). Exosomes in Cancer Liquid Biopsy: A Focus on Breast Cancer. Mol. Ther. Nucleic Acids.

[32] Chen G., Huang A.C., Zhang W., Zhang G., Wu M., Xu W., Yu Z., Yang J., Wang B., Sun H. (2018). Exosomal PD-L1 Contributes to Immunosuppression and Is Associated with Anti-PD-1 Response. Nature.

[33] Ogata-Kawata H., Izumiya M., Kurioka D., Honma Y., Yamada Y., Furuta K., Gunji T., Ohta H., Okamoto H., Sonoda H. (2014). Circulating Exosomal microRNAs as Biomarkers of Colon Cancer. PLoS ONE.

[34] Cheng C.-A., Hou K.-C., Hsu C.-W., Chiang L.-C. (2024). Ultrasensitive and High-Resolution Protein Spatially Decoding Framework for Tumor Extracellular Vesicles. Adv. Sci..

[35] Zhao Z., Yang Y., Zeng Y., He M. (2016). A Microfluidic ExoSearch Chip for Multiplexed Exosome Detection towards Blood-Based Ovarian Cancer Diagnosis. Lab. Chip.

[36] Heikal L.A., Hamdallah S.I., El-Habashy S.E., Ashour A.A., El-Moslemany R.M., El-Kamel A.H. (2026). Microfluidics for Separation, Detection, and Engineering of Extracellular Vesicles. Adv. Drug Deliv. Rev..

[37] Yadav A., Xuan Y., Sen C.K., Ghatak S. (2024). Standardized Reporting of Research on Exosomes to Ensure Rigor and Reproducibility. Adv. Wound Care.

[38] Buschmann D., Mussack V., Byrd J.B. (2021). Separation, Characterization, and Standardization of Extracellular Vesicles for Drug Delivery Applications. Adv. Drug Deliv. Rev..

[39] Kumar G., Alqahtani H. (2022). Deep Learning-Based Cancer Detection-Recent Developments, Trend and Challenges. Comput. Model. Eng. Sci..

[40] Khalifa M., Albadawy M. (2024). Using Artificial Intelligence in Academic Writing and Research: An Essential Productivity Tool. Comput. Methods Programs Biomed. Update.

[41] Heaton J. (2018). Ian Goodfellow, Yoshua Bengio, and Aaron Courville: Deep Learning: The MIT Press, 2016, 800 Pp, ISBN: 0262035618. Genet. Program. Evolvable Mach..

[42] LeCun Y., Bengio Y., Hinton G. (2015). Deep Learning. Nature.

[43] McKinney S.M., Sieniek M., Godbole V., Godwin J., Antropova N., Ashrafian H., Back T., Chesus M., Corrado G.S., Darzi A. (2020). Addendum: International Evaluation of an AI System for Breast Cancer Screening. Nature.

[44] Ardila D., Kiraly A.P., Bharadwaj S., Choi B., Reicher J.J., Peng L., Tse D., Etemadi M., Ye W., Corrado G. (2019). End-to-End Lung Cancer Screening with Three-Dimensional Deep Learning on Low-Dose Chest Computed Tomography. Nat. Med..

[45] Raufaste-Cazavieille V., Santiago R., Droit A. (2022). Multi-Omics Analysis: Paving the Path toward Achieving Precision Medicine in Cancer Treatment and Immuno-Oncology. Front. Mol. Biosci..

[46] Menden M.P., Wang D., Mason M.J., Szalai B., Bulusu K.C., Guan Y., Yu T., Kang J., Jeon M., Wolfinger R. (2019). Community Assessment to Advance Computational Prediction of Cancer Drug Combinations in a Pharmacogenomic Screen. Nat. Commun..

[47] Lambin P., Leijenaar R.T.H., Deist T.M., Peerlings J., De Jong E.E.C., Van Timmeren J., Sanduleanu S., Larue R.T.H.M., Even A.J.G., Jochems A. (2017). Radiomics: The Bridge between Medical Imaging and Personalized Medicine. Nat. Rev. Clin. Oncol..

[48] Bibault J.-E., Giraud P., Burgun A. (2016). Big Data and Machine Learning in Radiation Oncology: State of the Art and Future Prospects. Cancer Lett..

[49] Aliper A., Plis S., Artemov A., Ulloa A., Mamoshina P., Zhavoronkov A. (2016). Deep Learning Applications for Predicting Pharmacological Properties of Drugs and Drug Repurposing Using Transcriptomic Data. Mol. Pharm..

[50] Gawehn E., Hiss J.A., Schneider G. (2016). Deep Learning in Drug Discovery. Mol. Inform..

[51] Obermeyer Z., Emanuel E.J. (2016). Predicting the Future—Big Data, Machine Learning, and Clinical Medicine. N. Engl. J. Med..

[52] Topol E.J. (2019). High-Performance Medicine: The Convergence of Human and Artificial Intelligence. Nat. Med..

[53] Shin H., Choi B.H., Shim O., Kim J., Park Y., Cho S.K., Kim H.K., Choi Y. (2023). Single Test-Based Diagnosis of Multiple Cancer Types Using Exosome-SERS-AI for Early Stage Cancers. Nat. Commun..

[54] Tiwari A., Widodo, Krisnawati D.I., Tzou K.-Y., Kuo T.-R. (2026). Machine Learning for Extracellular Vesicles Enables Diagnostic and Therapeutic Nanobiotechnology. J. Nanobiotechnol..

[55] Hu Y., Yang X., Shu F., Jin L., Sarsaiya S., Han S., Chen J. (2026). Smart Exosomes: Multimodal Engineering Strategies for Logic-Gated and Stimuli-Responsive Precision Theranostics. Int. J. Pharm..

[56] Kim J., Yang J.D., Agopian V.G., Zhu Y., Tseng H.-R., You S. (2026). Computational Frameworks for Enhanced Extracellular Vesicle Biomarker Discovery. Exp. Mol. Med..

[57] Mathieu M., Névo N., Jouve M., Valenzuela J.I., Maurin M., Verweij F.J., Palmulli R., Lankar D., Dingli F., Loew D. (2021). Specificities of Exosome versus Small Ectosome Secretion Revealed by Live Intracellular Tracking of CD63 and CD9. Nat. Commun..

[58] Kawakami E., Tabata J., Yanaihara N., Ishikawa T., Koseki K., Iida Y., Saito M., Komazaki H., Shapiro J.S., Goto C. (2019). Application of Artificial Intelligence for Preoperative Diagnostic and Prognostic Prediction in Epithelial Ovarian Cancer Based on Blood Biomarkers. Clin. Cancer Res. Off. J. Am. Assoc. Cancer Res..

[59] Al-Remawi M., Aburub F., Al-Akayleh F., Abdel-Rahem R.A., Ali Agha A.S.A. (2025). Artificial Intelligence in Lipidomics: Advancing Biomarker Discovery, Pathway Analysis, and Precision Medicine. 2025 1st International Conference on Computational Intelligence Approaches and Applications (ICCIAA).

[60] Coudray N., Ocampo P.S., Sakellaropoulos T., Narula N., Snuderl M., Fenyö D., Moreira A.L., Razavian N., Tsirigos A. (2018). Classification and Mutation Prediction from Non–Small Cell Lung Cancer Histopathology Images Using Deep Learning. Nat. Med..

[61] Haenssle H.A., Fink C., Schneiderbauer R., Toberer F., Buhl T., Blum A., Kalloo A., Hassen A.B.H., Thomas L., Reader study level-I and level-II Groups (2018). Man against Machine: Diagnostic Performance of a Deep Learning Convolutional Neural Network for Dermoscopic Melanoma Recognition in Comparison to 58 Dermatologists. Ann. Oncol..

[62] Boehm K.M., Khosravi P., Vanguri R., Gao J., Shah S.P. (2022). Harnessing Multimodal Data Integration to Advance Precision Oncology. Nat. Rev. Cancer.

[63] Guo L., Du S., Gao S., Zhao R., Huang G., Jin F., Teng Y., Zhang L. (2022). Delta-Radiomics Based on Dynamic Contrast-Enhanced MRI Predicts Pathologic Complete Response in Breast Cancer Patients Treated with Neoadjuvant Chemotherapy. Cancers.

[64] Johannet P., Coudray N., Donnelly D.M., Jour G., Illa-Bochaca I., Xia Y., Johnson D.B., Wheless L., Patrinely J.R., Nomikou S. (2021). Using Machine Learning Algorithms to Predict Immunotherapy Response in Patients with Advanced Melanoma. Clin. Cancer Res..

[65] Chowell D., Yoo S.-K., Valero C., Pastore A., Krishna C., Lee M., Hoen D., Shi H., Kelly D.W., Patel N. (2022). Improved Prediction of Immune Checkpoint Blockade Efficacy across Multiple Cancer Types. Nat. Biotechnol..

[66] Hao X., Liu Z., Ma F., Li T., Liu C., Wang N., Guan J., He N., Liu J., Lu S. (2025). Exosome-Based Liquid Biopsy in Early Screening and Diagnosis of Cancers. Dose-Response.

[67] Welsh J.A., Goberdhan D.C.I., O’Driscoll L., Buzas E.I., Blenkiron C., Bussolati B., Cai H., Di Vizio D., Driedonks T.A.P., Erdbrügger U. (2024). Minimal Information for Studies of Extracellular Vesicles (MISEV2023): From Basic to Advanced Approaches. J. Extracell. Vesicles.

[68] Rai A., Huynh K., Cross J., Poh Q.H., Fang H., Claridge B., Duong T., Duarte C., Shaw J.E., Marwick T.H. (2025). Multi-Omics Identify Hallmark Protein and Lipid Features of Small Extracellular Vesicles Circulating in Human Plasma. Nat. Cell Biol..

[69] Simancas-Racines D., Román-Galeano N.M., Vásquez J.P., Jima Gavilanes D., Vijayan R., Reytor-González C. (2025). Liquid Biopsy and Multi-Omic Biomarkers in Breast Cancer: Innovations in Early Detection, Therapy Guidance, and Disease Monitoring. Biomedicines.

[70] Xu L., Li J., Gong W. (2025). Applications of Machine Learning-Assisted Extracellular Vesicles Analysis Technology in Tumor Diagnosis. Comput. Struct. Biotechnol. J..

[71] Lei Y., Fei X., Ding Y., Zhang J., Zhang G., Dong L., Song J., Zhuo Y., Xue W., Zhang P. (2023). Simultaneous Subset Tracing and miRNA Profiling of Tumor-Derived Exosomes via Dual-Surface-Protein Orthogonal Barcoding. Sci. Adv..

[72] Hsu C.-Y., Askar S., Alshkarchy S.S., Nayak P.P., Attabi K.A.L., Khan M.A., Mayan J.A., Sharma M.K., Islomov S., Soleimani Samarkhazan H. (2026). AI-Driven Multi-Omics Integration in Precision Oncology: Bridging the Data Deluge to Clinical Decisions. Clin. Exp. Med..

[73] Chen H., Liu H., Xing L., Fan D., Chen N., Ma P., Zhang X. (2025). Deep Learning-Driven Microfluidic-SERS to Characterize the Heterogeneity in Exosomes for Classifying Non-Small Cell Lung Cancer Subtypes. ACS Sens..

[74] Liangsupree T., Multia E., Riekkola M.-L. (2021). Modern Isolation and Separation Techniques for Extracellular Vesicles. J. Chromatogr. A.

[75] Veerman R.E., Teeuwen L., Czarnewski P., Güclüler Akpinar G., Sandberg A., Cao X., Pernemalm M., Orre L.M., Gabrielsson S., Eldh M. (2021). Molecular Evaluation of Five Different Isolation Methods for Extracellular Vesicles Reveals Different Clinical Applicability and Subcellular Origin. J. Extracell. Vesicles.

[76] Asleh K., Dery V., Taylor C., Davey M., Djeungoue-Petga M.-A., Ouellette R.J. (2023). Extracellular Vesicle-Based Liquid Biopsy Biomarkers and Their Application in Precision Immuno-Oncology. Biomark. Res..

[77] EV-TRACK Consortium, Van Deun J., Mestdagh P., Agostinis P., Akay Ö., Anand S., Anckaert J., Martinez Z.A., Baetens T., Beghein E. (2017). EV-TRACK: Transparent Reporting and Centralizing Knowledge in Extracellular Vesicle Research. Nat. Methods.

[78] Stordy B.P., Sepahi Z., Patrón G.D., Yang W., Goodson A.D., Blackadar C., Tavares A.J., Lin G., Malekjahani A., Ling B. (2025). The Binding Affinities of Serum Proteins to Nanoparticles. J. Am. Chem. Soc..

[79] Gao H., Zhan Y., Liu Y., Zhu Z., Zheng Y., Qian L., Xue Z., Cheng H., Nie Z., Ge W. (2025). Systematic Evaluation of Blood Contamination in Nanoparticle-Based Plasma Proteomics. EMBO Mol. Med..

[80] Latifi-Navid H., Mokhtari S., Taghizadeh S., Moradi F., Poostforoush-Fard D., Alijanpour S., Aghanoori M.-R. (2025). AI-Assisted Multi-OMICS Analysis Reveals New Markers for the Prediction of AD. Biochim. Biophys. Acta BBA-Mol. Basis Dis..

[81] Yu D., Li Y., Wang M., Gu J., Xu W., Cai H., Fang X., Zhang X. (2022). Exosomes as a New Frontier of Cancer Liquid Biopsy. Mol. Cancer.

[82] Li J., Wang A., Guo H., Zheng W., Chen R., Miao C., Zheng D., Peng J., Wang J., Chen Z. (2025). Exosomes: Innovative Biomarkers Leading the Charge in Non-Invasive Cancer Diagnostics. Theranostics.

[83] Ali Moussa H.Y., Manaph N., Ali G., Maacha S., Shin K.C., Ltaief S.M., Gupta V., Tong Y., Ponraj J., Salloum-Asfar S. (2022). Single Extracellular Vesicle Analysis Using Flow Cytometry for Neurological Disorder Biomarkers. Front. Integr. Neurosci..

[84] Cherukuri S.P., Kaur A., Goyal B., Kukunoor H.R., Sahito A.F., Sachdeva P., Yerrapragada G., Elangovan P., Shariff M.N., Natarajan T. (2025). Artificial Intelligence-Enhanced Liquid Biopsy and Radiomics in Early-Stage Lung Cancer Detection: A Precision Oncology Paradigm. Cancers.

[85] Yokoi A., Yoshida K., Koga H., Kitagawa M., Nagao Y., Iida M., Kawaguchi S., Zhang M., Nakayama J., Yamamoto Y. (2023). Spatial Exosome Analysis Using Cellulose Nanofiber Sheets Reveals the Location Heterogeneity of Extracellular Vesicles. Nat. Commun..

[86] Di Sario G., Rossella V., Famulari E.S., Maurizio A., Lazarevic D., Giannese F., Felici C. (2023). Enhancing Clinical Potential of Liquid Biopsy through a Multi-Omic Approach: A Systematic Review. Front. Genet..

